# Infections of *Cryptococcus* species induce degeneration of dopaminergic neurons and accumulation of *α*-Synuclein in *Caenorhabditis elegans*

**DOI:** 10.3389/fcimb.2022.1039336

**Published:** 2022-10-26

**Authors:** Thitinan Kitisin, Watcharamat Muangkaew, Passanesh Sukphopetch

**Affiliations:** Department of Microbiology and Immunology, Faculty of Tropical Medicine, Mahidol University, Bangkok, Thailand

**Keywords:** Parkinson’s disease, Parkinsonism, Cryptococcosis, dopaminergic neurons, *α*-synuclein accumulation, *Caenorhabditis elegans*

## Abstract

Cryptococcosis in the central nervous system (CNS) can present with motor declines described as Parkinsonism. Although several lines of evidence indicate that dopaminergic (DA) neuron degeneration and α-synuclein accumulation contribute to the hallmark of Parkinsonism and Parkinson’s disease (PD), little is known about cryptococcal infections associated with neuronal degeneration. In this study, the effects of *Cryptococcus neoformans* and *C. gattii* infections on dopaminergic neuron degeneration, α-synuclein accumulation, and lifespan in *Caenorhabditis elegans* were investigated. The results showed that cryptococcal infections significantly (*P*<0.05) induced DA neuron degeneration similar to a selective cathecholamine neurotoxin 6-hydroxydopamine (6-OHDA) in *C. elegans* (BZ555 strain) when compared to mock infected controls. Cryptococcal infections also significantly (*P*< 0.05) induced α-synuclein aggregation in *C. elegans* (NL5901 strain). Moreover, lifespan of the infected worms was significantly decreased (*P*<0.0001). In conclusion, DA neurodegeneration and α-synuclein accumulation are associated with lifespan reduction during cryptococcal infection in *C elegans*.

## Introduction

Cryptococcosis, is caused by infections of encapsulated basidiomycetous fungi of the genus *Cryptococcus*, is increasingly reported among acquired immunodeficiency syndrome (AIDS) patients and other immunosuppressed patients as well as in immunocompetent populations worldwide (Firacative et al., 2021). Environmental exposure to cryptococci is common as these pathogenic yeasts are ubiquitously found in soil, water, certain contaminated foods, and pigeon droppings (Malik et al., 2003). Currently, most cases are caused by *Cryptococcus neoformans*, however, infections of *C. gattii* are also reported (Olszewski et al., 2010). Infections of cryptococci are primarily caused by inhalation of infectious propagules (e.g., poorly encapsulated yeast cells and/or basidiospores) from contaminated reservoirs to the pulmonary alveoli, however, severe meningoencephalitis is the commonest clinical presentation (Stott et al., 2021). Cryptococci process a unique ability to penetrate through the blood brain barrier (BBB) and colonized the brain, which conferred a strong neurotropism (Esher et al., 2018). Cryptococcal infections that damage central nervous system (CNS) are highly lethal and cause several neurological disorders depending on the site of cerebral infections (Colombo and Rodrigues, 2015). Several clinical studies in humans and animal models have revealed the pattern of neurological disorders after cryptococcal infections include meningitis, encephalitis, meningoencephalitis, ventriculitis, increased intracranial pressure (ICP), and Parkinsonism (Camargos et al., 2006; Colombo and Rodrigues, 2015). Interestingly, Parkinsonian features during cryptococcal meningoencephalitis have been reported over the last three decades (Wszolek et al., 1988). Nevertheless, understanding the pathogenesis of cryptococcosis-induced CNS damage, particularly Parkinsonian symptoms, is limited and poorly investigated.

Patients with cryptococcal-induced Parkinsonism are often presented with prominent clinical characteristics including bradykinesia, tremor, rigidity, and postural instability. Postmortem brain examination has confirmed the fungal burden located at the substantia nigra and in basal ganglia suggesting the root-cause of Parkinsonism (Wszolek et al., 1988; Pedroso and Barsottini, 2012; Nelles et al., 2022). Degeneration of dopaminergic (DA) neurons of the substantia nigra and α-synucleinopathies are the common denominators of Parkinsonism during aging (Dickson, 2012). However, pathological investigation of the infectious yeast cryptococci to the degeneration of DA neurons,α-synuclein accumulation, and longevity is still poorly determined.

It is well known that fungal contamination in routine laboratory of cell culture can rapidly kill mammalian cells *in vitro.* However, cryptococcal yeast cells have little cytotoxic effect when co-cultured with animal cells *in vitro* suggesting that cryptococcal infections are not involved with tissue necrosis as seen in those infections caused by *Aspergillus* spp. (Casadevall et al., 2018) or *Scedosporium* spp. (Cortez et al., 2008). Moreover, it is even more difficult to isolate DA neurons from mammalian brains for studying cryptococcosis *in vitro* (Park et al., 2020). Murine models of *Cryptococcus* meningoencephalitis have been successfully developed but do not mimic to cryptococcal-induced Parkinsonism (Thompson et al., 2012; Sabiiti et al., 2012). Several alternative cryptococcosis invertebrate models have been introduced including *Caenorhabditis elegans* (Mylonakis et al., 2002; Mylonakis et al., 2004), *Drosophila melanogaster* (Apidianakis et al., 2004), and *Galleria mellonella* (Mylonakis et al., 2005). In a previous study, our laboratory showed that both *C. neoformans* ATCC#32045 and *C. gattii* ATCC#56992 can reduce the lifespan of *C. elegans* by regulating the insulin/insulin-like growth factor-1 (IGF-1) signaling (IIS) pathway and DAF-16 has been shown to play a central role during cryptococcal infections (Kitisin et al., 2022). Although *C. elegans* has been extensively used as a model to screen targeted drugs for anti-Parkinson effects (Chalorak et al., 2018; Malaiwong et al., 2019), however, the study of *C. elegans* neurodegeneration caused by pathogenic fungal infections has been limited.

The *C. elegans* model of neurodegeneration exhibits several biological advantages, as its simple anatomy, microscopic transparency, short lifespan, facile genetics, and eased ethical constraints, which are a great challenge when using vertebrate models (Teschendorf and Link, 2009). In general, *C. elegans* has a total of 959 somatic cells including 302 neuronal cells (Chalorak et al., 2018). Interestingly, *C. elegans* has exactly 8 DA neurons (ADEL, ADER, CEPDL, CEPDR, CEPVL, CEPVR, PDEL, and PDER), which are structurally and functionally similar to human DA neurons (Sulston et al., 1975; Maulik et al., 2017). Six DA neurons are located at the anterior part of the body as 4 anterior cephalic (CEP) and 2 anterior deirid (ADE) neurons and the 2 remaining posterior DA neurons are known as posterior deirid (PDE) neurons (Harrington et al., 2010). In the present study, we used transgenic *C. elegans* (BZ555 and NL5901 strains) to investigate the degeneration of DA neurons caused by cryptococcal infections. The *C. elegans* strain BZ555 has been created to localize the DA neurons that express green fluorescent protein (GFP) specifically tagged to the *C. elegans dat-1* gene (DA transporter gene) (Nass et al., 2002). By chemically exposing the *C. elegans* DA neurons to neurotoxin 6-hydroxydopamine (6-OHDA), DA degeneration can be demonstrated (Offenburger et al., 2018). In addition, α-synucleinopathy can be observed using *C. elegans* strain NL5901, which express yellow fluorescent protein (YFP) under the specific and strong promotion of *unc-54* gene (van Ham et al., 2008). Moreover, the lifespan of worms after cryptococcal infections was also determined. Thus, the present study demonstrates the degeneration of DA neurons caused by cryptococcal infections and provides an alternative model of *C. elegans* for studying the pathogenesis of infectious etiologies of Parkinsonism.

## Materials and methods

### Strains and culture conditions

*C. neoformans* (ATCC#32045) and *C. gattii* (ATCC#56992) strains were obtained from the American Type Culture Collection (ATCC). Fungal stock cultures were stored at −80°C in 25% glycerol until use. Yeasts were maintained on Yeast-Peptone-Dextrose (YPD) agar (Oxoid, Hampshire, UK) at 30°C unless otherwise specified (Tang et al., 2005). *C. elegans* transgenic BZ555 [egIs1 [dat-1p::GFP]] and NL5901 [pkIs2386 [unc-54p::α-synuclein:YFP + unc-119(+)]] strains were obtained from the *Caenorhabditis* Genetics Center (CGC, USA) of the University of Minnesota. *C. elegans* were maintained as described previously (Kitisin et al., 2022). *Escherichia coli* OP50 was obtained from the CGC and used as a food source for *C. elegans*. *E. coli* OP50 were grown in Luria Broth (LB, BD) by shaking overnight at 37°C, collected by centrifugation at 5,000 × g for 10 min, and diluted to OD = 0.1 using sterile M9 buffer. An alkaline hypochlorite treatment (12% NaClO and 10% 1 M NaOH) of gravid hermaphrodites was used to obtain synchronized L1 larvae and maintained on solid nematode growth media (NGM) fed with *E. coli* OP50 at 25°C until the L4 stage (3-day-old worms). All experiments used the L4 worms as day 0 of the experiments to control the reproductive systems in *C. elegans* (Oh et al., 2022).

### Infections of *C. elegans* by *Cryptococcus* species and 6-OHDA treatment

*C. neoformans* and *C. gattii* strains were inoculated into 2 ml of YPD broth and grown at 30°C for 48 h. Then, 10 μl of fungal yeast cells was spread on 35-mm tissue-culture plate containing Brain Heart Infusion (BHI, Difco) agar supplemented with 150 μM of 5-fluoro-2′-deoxyuridine (FDdR, Sigma Aldrich) to inhibit the development of worm’s progeny (Mylonakis et al., 2002). Moreover, 50 μg/ml of kanamycin (Sigma Aldrich) was added to the BHI plates to prevent the growth of *E. coli* OP50 that might have been carried over during the transfer of worms to the yeast-containing BHI plates (Pukkila-Worley et al., 2009). To perform an infection assay, age-synchronized L4 worms (BZ555 strain) were transferred from a lawn of *E. coli* OP50 on NGM plates to the prepared yeast cells on BHI plates and incubated at 25°C for 72 h. Worms that fed with *E. coli* OP50 were used as mock-infected controls. To chemically ablate the worms’ dopaminergic neurons, control BZ555 worms grown for 24 h on *E. coli* OP50/NGM/FUdR plates were collected and washed with M9 buffer. Control worms were incubated with 50 mM of 6-hydroxy-dopamine (6-OHDA, Sigma Aldrich) and 10 mM ascorbic acid (Sigma Aldrich) (Nass et al., 2002). The assay solution (1 ml) was gently mixed in every 10 min at 20°C for 1 h. Then, the 6-OHDA treated worms were washed three times with M9 buffer and transferred to the new *E. coli* OP50/NGM/FUdR plates and incubated at 25°C for 48 h and served as a positive control for DA degeneration. Control BZ555 worms incubated on *E. coli* OP50/NGM/FUdR plates without 6-OHDA treatment were used as a positive control. Each experimental condition was performed in triplicate.

### Quantification of *C. elegans* DA degeneration

Degeneration of DA neurons was observed in BZ555 worms after infection with either *C. neoformans*, *C. gattii* or treated with 6-OHDA as described previously. The worms were then washed three times with M9, immobilized with a few drops of 100 mM sodium azide (NaN_3_) on 1% agarose pads, and finally enclosed by a cover slip. Fifty of worms were photographed using a Zeiss Axio Imager fluorescence microscope (ZEISS, Germany) using an LED source and a GFP filter. The fluorescence intensity was measured using ImageJ software (National Institute of Health, NIH, Bethesda, MD, USA).

### Evaluation of *C. elegans* lifespan

The mean lifespan of BZ555 worms was determined by feeding on *E. coli* OP50, *E. coli* OP50/6-OHDA, *C. neoformans* or *C. gattii* as described (Mylonakis et al., 2002; Chalorak et al., 2018). In brief, fifty synchronized L4 of BZ555 were transferred onto yeast-containing BHI/FUdR plates and incubated at 25°C. Worms cultured on *E. coli* OP50 alone were used as a control, whereas worms incubated with 6-OHDA were used as a positive control. The animals remained on the experimental plates for the remainder of their life spans. Number of live and dead worms were counted every 24 h until all the worms died. Failure to respond a stimulation using a platinum wire pick or no sign of pharyngeal pumping movement were considered dead worms. Alternatively, worms cultured on BHI plates were transferred to new plates every 24 - 48 h to remove the presence of progeny. Worms that suffered from developmental defects, internal hatching, or those that burrowed, or crawled off the plates were censored from the analysis. Summation of worms in triplicates were used to statistically calculate the lifespan excluding the censored worms.

### Quantification of *C. elegans* α-synuclein accumulation

Accumulation of α-synuclein was observed in NL5901 worms after infection with *C. neoformans* and *C. gattii* as described previously with some modifications (Chalorak et al., 2018; Kitisin et al., 2022). Briefly, age-synchronized L4 NL5901 worms were transferred onto yeast-containing BHI/FUdR plates and incubated at 25°C for 72 h. Worms cultured on *E. coli* OP50 were used as a control. After 72 h, the worms were washed with M9 buffer and immobilized with NaN_3_ on an agar pad. The fluorescence intensity of fifty worms in each condition was determined using fluorescence microscope and quantified by using ImageJ software.

### Statistical analysis

Data were expressed as mean ± SD. All the experiments were performed in triplicate. The Kaplan-Meier lifespan analysis and log-rank test were used to calculate the mean lifespan and *P*-values, respectively. In the other experiments, one way or two-way ANOVA was conducted to test the differences. Statistical analyses were determined using GraphPad Prism software (GraphPad Software, Inc., San Diego, California, USA). Levels of significance were indicated as *P < 0.05; **P < 0.01; ***P < 0.001; ****P < 0.0001; NS, not significant (*P* > 0.05).

### Ethical statement

This study was performed under the Animals for Scientific Purposes Act, B.E. 2558 (A.D. 2015), Thailand. *C. elegans* strains were provided by the CGC. *C. elegans* protocols were approved by the Faculty of Tropical Medicine – Animal Care and Use Committee (FTM‐ACUC), Mahidol University, Thailand, No. FTM024-2020. Safe handling of *C. neoformans* (ATCC#32045) and *C. gattii* (ATCC#56992) strains were approved by Institutional Biosafety Committee, Faculty of Tropical Medicine, Mahidol University, Thailand, Submission No. FTM-IBC-20-09.

## Results

### Infections of *C. neoformans* and *C. gattii* induced DA neuronal degradation in *C. elegans*

**Figure 1:**
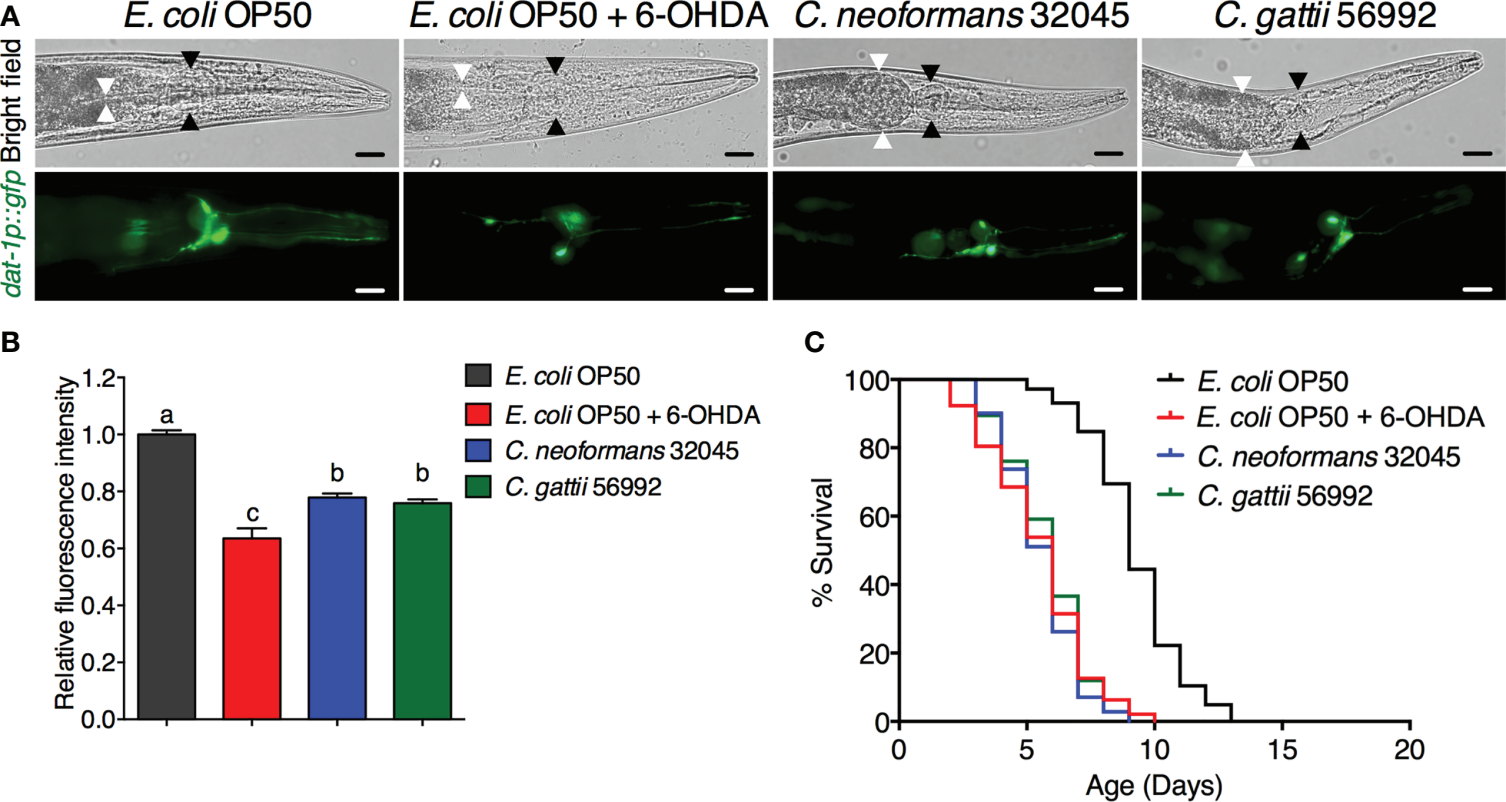
Infections of *C neoformans* and *C gattii* induced DA neuronal degradation are similar to 6-OHDA toxicity in *C elegans*. **(A)** The BZ555 worms that expressing GFP on DA neurons were exposed to either *C neoformans* (ATCC#32045), *C gattii* (ATCC#56992) or 6-OHDA. Upon infection, the cryptococcal yeast cells were seen at the distal area of the abnormally distended gastrointestinal tract of worms. Black arrowheads indicate the worm pharyngeal grinder. White arrowheads indicate the intestinal lumen of the nematode. Worms from all experiments were photographed alive. Scale bar = 20 μm. **(B)** Quantitation of GFP expression in DA neurons was measured by using ImageJ software. The data represented as mean ± SD (n = 30). ^a–c^Indicated statistically significant differences between experimental conditions (*P* < 0.05) by two-way ANOVA. **(C)** Percent survival of worms fed with *E coli* OP50/6-OHDA, *C neoformans* or *C gattii.* Worms that fed with *E coli* OP50 alone were served as mock-infected controls (n = 50). Survival was analyzed using Kaplan-Meier lifespan analysis. *P*-value was calculated using log-rank test. Mean lifespan differences between each condition was summarized in **Table 1**.

The DA neurons were evaluated by analyzing the mean fluorescence intensity of GFP expressions of BZ555 strain. The ADE and CEP neurons were highly degenerated after treating with 6-OHDA and showed partial loss of GFP to approximately 63.52 ± 3.54% (*P* < 0.05) when compared to controls (100 ± 1.49%, **Figures 1A, B**). Upon cryptococcal infection, we found an accumulation of *C. neoformans* and *C. gattii* yeast cells and the worms exhibited abnormally distended gastrointestinal tracts, which were surrounded by 6 DA neurons 4 anterior cephalic (CEP) and 2 anterior deirid (ADE) neurons (**Figure 1A**) (Harrington et al., 2010). Interestingly, GFP expression of DA neurons (ADE and CEP) in BZ555 worms infected with *C. neoformans* or *C. gattii* were significantly reduced to 77.88 ± 1.39% (*P* < 0.05) and 75.90 ± 1.30% (*P* < 0.05), respectively (**Figures 1A, B**). Degeneration of DA neurons caused by 6-OHDA treatment reduced the lifespan of BZ555 worms from 9.26 ± 1.79 days to 5.48 ± 1.96 days (-40.82%, *P* < 0.0001, **Figure 1C**, **Table 1**). Moreover, degeneration of DA neurons caused by the infections of *C. neoformans* or *C. gattii* also reduced the lifespan to 5.51 ± 1.47 days (-40.49%, *P* < 0.0001) or 5.76 ± 1.58 days (-37.80%, *P* < 0.0001) (**Figure 1C**, **Table 1**). Thus, cryptococci-induced DA degenerations may reduce lifespan in *C. elegans*.

**Table 1:** Mean lifespan of *C. elegans* strain BZ555 and NL5901 upon *C. neoformans* 32045 or *C. gattii* 56992 infections.

Strain	Condition	Mean	SD	SE	Median	Max	n	*P*-value		%
BZ555	+ *E. coli* OP50	9.26	1.79	0.15	9.00	13.00	144			
	+ *E. coli* OP50 + 6-OHDA	5.48	1.96	0.16	6.00	10.00	143	< 0.0001	****	-40.82%
	+ *C. neoformans* 32045	5.51	1.47	0.12	6.00	9.00	141	< 0.0001	****	-40.49%
	+ *C. gattii* 56992	5.76	1.58	0.13	6.00	9.00	142	< 0.0001	****	-37.80%
NL5901	+ *E. coli* OP50	6.25	1.31	0.11	6.00	9.00	145			
	+ *C. neoformans* 32045	3.74	1.34	0.11	4.00	6.00	144	< 0.0001	****	-40.16%
	+ *C. gattii* 56992	3.61	1.37	0.11	4.00	6.00	145	< 0.0001	****	-42.24%

**Figure 2:**
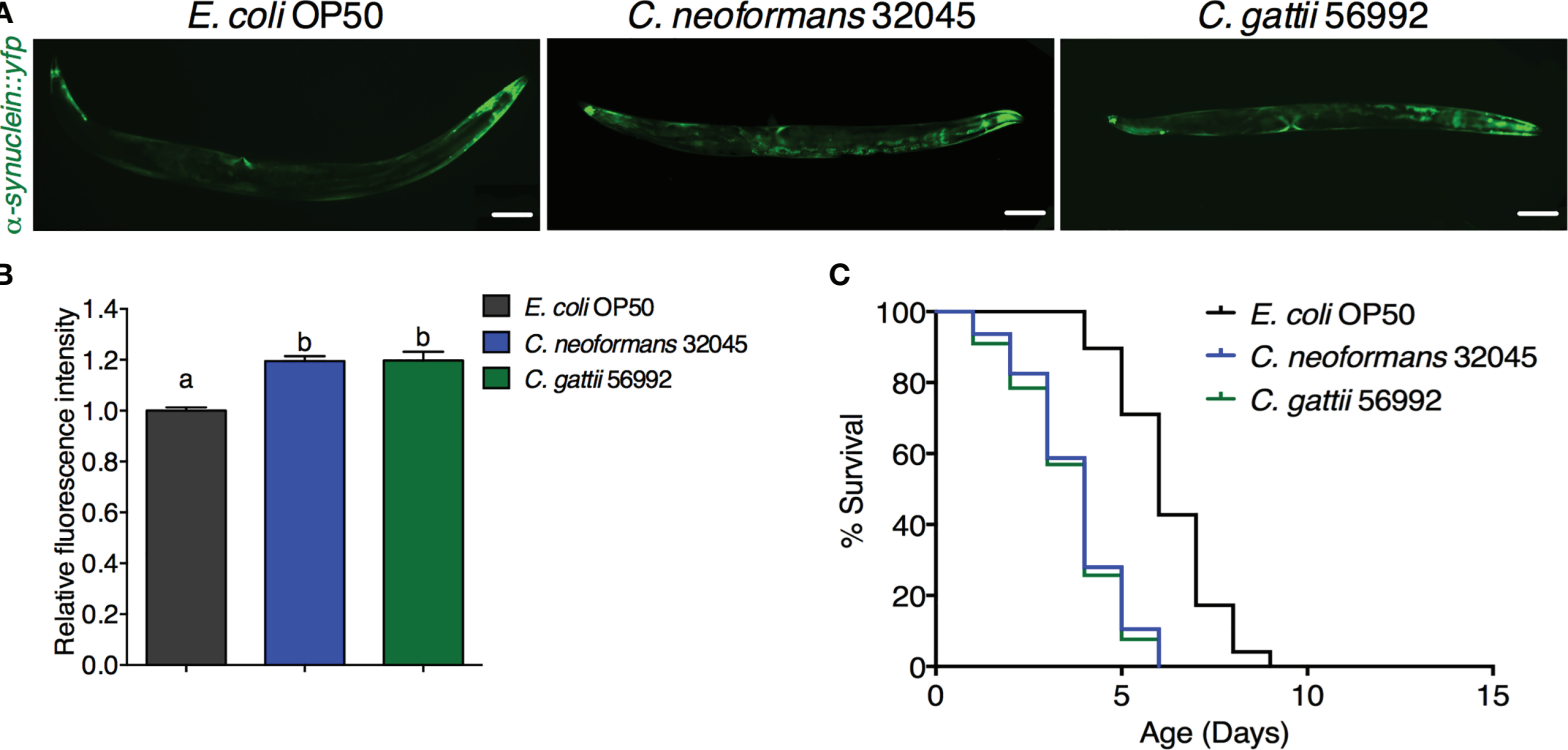
Infections of *C neoformans* and *C gattii* induced α-synuclein accumulation in *C elegans*. **(A)** *C elegans* strain NL5901 expressing YFP in muscle cells were exposed to either *C neoformans* (ATCC#32045) or *C gattii* (ATCC#56992) (scale bar = 200μm). **(B)** Quantitation of YFP expression in muscle cells was measured by using ImageJ software. The data represented as mean ± SD (n = 30). ^a–b^Indicated statistically significant differences between experimental conditions (*P* < 0.05) by two-way ANOVA. **(C)** Percent survival of worms fed with *C neoformans* or *C gattii.* Worms that fed with *E coli* OP50 alone were served as mock-infected controls (n = 50). Survival was analyzed using Kaplan-Meier lifespan analysis. *P*-value was calculcated using log-rank test. Mean lifespan in each condition was summarized in **Table 1**.

Means, standard deviation (SD), standard error (SE), median, and maximum of lifespan were shown in days. Total worms (n) were represented as summation of worms in triplicates (with censored worms excluded). The lifespan data were analyzed using the log‐rank test and P‐values for each individual experiment were shown when compared to corresponding control as *P < 0.05; **P < 0.01; ***P < 0.001; ****P < 0.0001; NS, not significant (P > 0.05). Results presented in **Figures 1C** and **2C**.

### Infections of *C. neoformans* and *C. gattii* induced the α-synuclein accumulation in *C. elegans*

The accumulation of human α-synuclein protein was evaluated by analyzing the mean fluorescence intensity of YFP expression in the muscle cells of the NL5901 strain. The level of α-synuclein was higher in *C. neoformans* or *C. gattii* infected worms (119.47 ± 1.98% or 119.75 ± 3.40%, respectively, *P* < 0.05) when compared to controls (100 ± 1.29%, **Figures 2A, B**). Accumulation of human α-synuclein protein caused by the infections of *C. neoformans* or *C. gattii* also reduced the lifespan from 6.25 ± 1.31 days to 3.74 ± 1.34 days (-40.16%, *P* < 0.0001) or 3.61 ± 1.37 days (-42.24%, *P* < 0.0001) (**Figure 2C**, **Table 1**). Thus, cryptococci-induced α-synuclein accumulation may reduce lifespan in *C. elegans*.

## Discussion

Dopaminergic neurons (DA) can be identified in rodent and primate brain sections by using immunolabeling techniques or using positron emission tomography *in vivo* (Gaspar et al., 1992). In the laboratory, cell-specific markers of the presynaptic dopamine transporter (DAT) are frequently used to experimentally reveal DA neurons *in vivo.* However, expression of DATs in mammalian neurons are greatly distributed throughout genomic DNA. Furthermore, mammalian DA neurons are located deep in the ventral midbrain and basal ganglia of the CNS causing a great challenge to study these neurons only in fully developed, intact adult brain (Hegarty et al., 2013). To overcome these limitations, all eight DA neurons can be revealed in *C. elegans* strain BZ555 by using only ~ 0.7 kb of 5′ flanking DAT-1 genomic sequence targeted to the reporter genes e.g., GFP. A previous study has shown that *C. elegans* DA neurons can be chemically degenerated by the treatment of 6-OHDA and used as a *C. elegans* model of Parkinson’s disease (Nass et al., 2002). Thus, we employed this transgenic *C. elegans* strain (BZ555) to visualize the DA neurons in a living animal. We previously reported that *C. neoformans* and *C. gattii* can accumulate and kill the wildtype (N2) *C. elegans* (Kitisin et al., 2022). In the present study, we found that accumulation of *C. neoformans* and *C. gattii* yeast cells in the distended gastrointestinal tract was anatomically close to 6 DA neurons (**Figures 1A, B**) (Harrington et al., 2010). Moreover, we found that infections of *C. neoformans* and *C. gattii* induced the degeneration of DA neurons and accelerated aging similarly to the 6-OHDA treatment (**Figure 1C**). These observations have led to the suggestion that invasive cryptococcal infections can induce cellular cytotoxicity and CNS tissue compression leading to the death of DA neurons as seen our present study and in patients with cryptococcal meningoencephalitis associated with prominent features of Parkinsonism (Wszolek et al., 1988; Pedroso and Barsottini, 2012; Nelles et al., 2022). Therefore, *C. elegans* can be used as a model to investigate the pathogenesis of cryptococcal infection that induces DA neuronal degeneration related to Parkinsonian symptoms.

Another hallmark of Parkinson’s disease is the accumulation of α-synuclein in the aging brain (Stefanis, 2012). A previous study has demonstrated that expression of human α-synuclein fused YFP protein *via unc-54* promoter in *C. elegans* strain NL5901 can be increasingly accumulated in age-dependent manner. Moreover, these inclusions of α-synuclein were structurally similar to human pathological inclusions in PD patients (Lakso et al., 2003). Therefore, we employed this transgenic *C. elegans* strain (NL5901) to visualize the accumulation of human α-synuclein in a living animal. We have previously reported that infections of *C. neoformans* and *C. gattii* can accelerate the aging process and reduce the lifespan of *C. elegans* involving with IIS/DAF-16 pathway (Kitisin et al., 2022). A recent study has also reported that accumulation of aggregated human α-synuclein inclusions was strongly associated with the activation of DAF-16 suggesting the relationship between the IIS pathway and PD (Haque et al., 2020). In the present study, we found that cryptococcal infections significantly increased human α-synuclein accumulation higher than mock infected controls, which associated with a reduction of infected worm’s lifespan (**Figure 2**). These observations suggest that chronic cryptococcal infections can induce the accumulation of pathological α-synuclein inclusions, which then accelerate neuronal loss as seen in patients with persistent Cryptococcal brain infection (Wingfield et al., 2014; Beardsley et al., 2015). Further study is required to elucidate the molecular mechanisms of cryptococcal induced DA degeneration and accumulation of α-synuclein during chronic infections in *C. elegans*.

## Conclusion

Infections of *C. neoformans* and *C. gattii* robustly degenerated DA neurons, increased the accumulation of α-synuclein, and reduced the lifespan of *C. elegans*. Nevertheless, our study provides an alternative *C. elegans* infection model of cryptococcal-induced neurodegeneration, which may be beneficial for novel therapeutic screening to prevent CNS damage against cryptococcal infections.

## Data availability statement

The original contributions presented in the study are included in the article. Further inquiries can be directed to the corresponding author.

## Author contributions

TK and PS: Designed the study. TK: Carried out the majority of the experiments, analyzed the data, and wrote the manuscript. WM: Assisted in *C. elegans* and cryptococcal cultures and maintenances. TK and PS: Reviewed and revised the manuscript, contributed to funding acquisitions. All authors read and approved the final version of this manuscript. All authors contributed to the article and approved the submitted version.

## Funding

This work was funded by Mahidol University (Contract no. A22/2564) to TK; Mahidol University (Basic Research Fund: fiscal year 2021) and Health Systems Research Institute (Grant number: HSRI 64-051) to PS.

## Acknowledgments

*Caenorhabditis elegans* strains used in this study were provided by the *Caenorhabditis* Genetics Center (CGC) of the University of Minnesota, which is funded by the National Institutes of Health (NIH)—Office of Research Infrastructure Programs (P40 OD010440). The authors would like to thank the Central Instrument Facility Unit, Faculty of Tropical Medicine, Mahidol University, Thailand for the use of fluorescence microscopy.

## Conflict of interest

The authors declare that the research was conducted in the absence of any commercial or financial relationships that could be construed as a potential conflict of interest.

## Publisher’s note

All claims expressed in this article are solely those of the authors and do not necessarily represent those of their affiliated organizations, or those of the publisher, the editors and the reviewers. Any product that may be evaluated in this article, or claim that may be made by its manufacturer, is not guaranteed or endorsed by the publisher.
